# Feminizing and masculinizing gender-affirming hormone therapy have distinct effects on fibrinogen γ’

**DOI:** 10.1016/j.rpth.2026.107026

**Published:** 2026-10-15

**Authors:** Mette Bøgehave, Dorte Glintborg, Louise Lehmann Christensen, Nadja Bødker Pedersen, Guy T'Sjoen, Martin den Heijer, Marianne Skovsager Andersen, Else-Marie Bladbjerg

**Affiliations:** 1 Department of Clinical Biochemistry, Unit for Thrombosis Research, University Hospital of Southern Denmark, Esbjerg, Denmark; 2 Department of Regional Health Research, University of Southern Denmark, Esbjerg, Denmark; 3 Department of Endocrinology, Odense University Hospital, Odense, Denmark; 4 Department of Clinical Research, University of Southern Denmark, Odense, Denmark; 5 Department of Endocrinology and Center for Sexology and Gender, Ghent University Hospital, Ghent, Belgium; 6 Department of Endocrinology and Metabolism & Center of Expertise on Gender Dysphoria, Amsterdam UMC, Amsterdam, the Netherlands

**Keywords:** estradiol, fibrinogen, testosterone, thrombosis, transgender persons

## Abstract

**Background:**

Fibrinogen γ’ is an alternatively spliced fibrinogen isoform. Higher fibrinogen γ’ levels are associated with arterial thrombosis and lower levels with venous thrombosis. Feminizing gender-affirming hormone therapy (GAHT) increases venous thrombosis risk, whereas masculinizing GAHT increases arterial thrombosis risk. How GAHT affects fibrinogen γ’ is unknown.

**Objectives:**

We aimed to investigate changes in fibrinogen γ’ levels after 12 months of feminizing or masculinizing GAHT.

**Methods:**

Fibrinogen γ’ and total fibrinogen were measured at baseline and after 12 months of GAHT in 245 transgender women and 318 transgender men (cohort study). Feminizing GAHT included oral or transdermal estradiol combined with cyproterone acetate (CPA) (3 groups). Masculinizing GAHT included intramuscular or transdermal testosterone with or without progestin (7 groups). Fibrinogen γ’ was measured by ELISA and total fibrinogen by nephelometry. Relative fibrinogen γ’ levels were calculated as the fibrinogen γ’/total fibrinogen ratio.

**Results:**

Fibrinogen γ’ increased during feminizing GAHT (588 vs 628 μg/mL, *P* < .001), while relative fibrinogen γ’ levels decreased (0.23 vs 0.22, *P* < .01). The increase in fibrinogen γ’ was most pronounced with oral estradiol plus CPA (*P* < .02), while the decrease in relative fibrinogen γ’ levels was comparable between treatment groups of feminizing GAHT. Fibrinogen γ’ decreased during masculinizing GAHT (698 vs 660 μg/mL, *P* < .001), while relative fibrinogen γ’ levels remained unchanged. No between-group differences were observed for masculinizing GAHT.

**Conclusion:**

Feminizing and masculinizing GAHT for 12 months had opposite effects on fibrinogen γ’ levels. Only the reduction in relative fibrinogen γ’ levels during feminizing GAHT may contribute to increased venous thrombosis risk.

## Essentials

• Fibrinogen γ’ is a variant of fibrinogen linked to thrombotic risk.
• We studied fibrinogen γ’ levels before and after 12 months of gender-affirming hormone therapy.
• Feminizing therapy (*n* = 245) increased absolute fibrinogen γ’ levels but decreased relative levels.
• Masculinizing therapy (*n* = 318) decreased absolute fibrinogen γ’ levels.

## 1 Introduction

Fibrinogen is a key determinant of thrombus formation. Fibrinogen is a structurally heterogeneous protein that exists in multiple molecular forms arising from genetic variation, alternative splicing, and posttranslational modifications [1]. Fibrinogen γ’ is an alternatively spliced fibrinogen isoform with a distinct C-terminus of the γ-chain [2]. Fibrinogen γ’ accounts for 10% to 25% of circulating fibrinogen, although reported levels vary between studies and assays [3], [4], [5]. Observational studies have linked fibrinogen γ’ to thrombotic disease, with higher levels reported in arterial thrombotic disorders [6], [7], [8], [9] and lower levels in venous thrombosis [10], [11], [12], although the mechanisms remain incompletely understood [6], [10], [12]. Fibrinogen γ’ binds thrombin and factor XIII with high affinity, thereby modulating fibrin formation and clot architecture [13], [14]. Experimental studies have shown that increased fibrinogen γ’ is associated with a prothrombotic fibrin clot structure, including reduced fiber diameter, and more lysis-resistant clots [15], [16], [17]. In addition, fibrinogen γ’ inhibits thrombin-mediated activation of coagulation factor V and enhances sensitivity to activated protein C, thereby exerting anticoagulant effects [18], [19].

Transgender and gender-diverse individuals (for simplicity, also referred to as transgender individuals) experience incongruence between their gender identity and sex assigned at birth. Gender-affirming hormone therapy (GAHT) is administered to induce physical characteristics aligned with gender identity. Feminizing GAHT has been associated with increased venous thrombotic risk, although increased risk of arterial thrombosis has also been reported, whereas masculinizing GAHT has primarily been linked to arterial cardiovascular events and less consistently with venous thrombosis [20], [21], [22]. Feminizing GAHT typically consists of estradiol (E) combined with antiandrogens, whereas masculinizing GAHT consists of testosterone (T), resulting in markedly increased circulating estrogen or T levels. GAHT is known to influence hepatic protein synthesis and circulating hemostatic factors, including fibrinogen [23], [24], [25]. Thus, 12 months of feminizing GAHT were associated with increased fibrinogen and prothrombotic fibrin clot properties following feminizing GAHT, and the opposite pattern was observed with masculinizing GAHT [25]. These changes in fibrin clot properties were only partly explained by changes in total fibrinogen concentrations [25], suggesting that GAHT may affect the molecular form of fibrinogen.

To our knowledge, the effect of GAHT on fibrinogen γ’ levels is unknown. We evaluated how 12 months of GAHT in different administration forms affect fibrinogen γ’ levels in a large prospective cohort study of transgender people. Given the increased risk of venous thrombosis associated with feminizing GAHT and the increased risk of arterial thrombosis associated with masculinizing GAHT, together with the reported opposite associations of fibrinogen γ’ levels with venous and arterial thrombosis, we hypothesize that fibrinogen γ’ levels decrease during feminizing GAHT and increase during masculinizing GAHT.

## 2 Methods

### 2.1 Participants and study design

This study is part of the European Network for the Investigation of Gender Incongruence. The study design, eligibility criteria, characteristics of participants, and ethical approval have been described in detail elsewhere [25], [26], [27], [28]. In brief, the study comprised 270 transgender women and 348 transgender men aged >17 years who visited gender identity clinics in Amsterdam and Ghent between April 2012 and March 2020, with blood samples collected at baseline and after 12 months of GAHT. Participants using estrogen-containing oral contraceptives or antiandrogens other than cyproterone acetate (CPA) were excluded. No participants received anticoagulant or antifibrinolytic therapy or underwent gender-affirming genital surgery during follow-up.

A detailed description of the treatment protocol is available elsewhere [25], [26]. In brief, feminizing GAHT consisted of E combined with the antiandrogen CPA, administered either orally, transdermally, or in combination (3 groups). Masculinizing GAHT consisted of T given as intramuscular injections (undecanoate or esters), as transdermal gel, or in combination with progestins (G) (7 groups) (Table 1 [25], [26]).

**Table 1:** Baseline characteristics and gender-affirming hormone therapy in transgender women and transgender men.

Transgender	*N* (Ams/Ghe)	Age (y)	Smokers (%)	Gender-affirming hormone therapy	Group name
Women	270 (182/88)	25 (21; 35)	20	Total estradiol group	E total
	175 (100/75)	23 (20; 28)	20	Estradiol valerate 2-6 mg/d and CPA 10-50 mg/d	E oral + CPA
	77 (68/9)	38 (27; 44)^a^	22	Estradiol patches 50-150 μg/d and CPA 10-50 mg/d	E transdermal + CPA
	18 (14/4)	27 (22; 43)	11	Estradiol valerate/patches^b^ and CPA 10-50 mg/d	E oral/transdermal + CPA
Men	348 (223/125)	21 (19; 26)	24	Total testosterone group	T total
	85 (27/58)	22 (19; 26)	24	Testosterone intramuscular undecanoate 1000 mg/10-12 wk	T IM undecanoate
	50 (50/0)	21 (19; 25)	24	Testosterone intramuscular ester 125-250 mg/2-3 wk	T IM ester
	45 (45/0)	22 (20; 27)	22	Testosterone gel 25-75 mg/d	T transdermal
	39 (38/1)	21 (20; 26)	28	Testosterone undecanoate/ester/gel^b^	T mixed
	37 (20/17)	21 (19; 28)	27	Testosterone undecanoate/ester/gel^b^ and progestin	T mixed + G
	70 (24/46)	21 (19; 24)	26	Baseline progestin + testosterone undecanoate/ester/gel^b^	G_0 + T mixed
	22 (19/3)	22 (19; 24)	14	Baseline progestin + testosterone undecanoate/ester/gel^b^ + progestin	G_0 + T mixed + G

Age is presented as median (25;75 percentile) and was compared between hormone groups using a Kruskal–Wallis test. Smoking is presented as current smokers as a percentage of the total group and was compared between hormone groups using a chi-squared test.

Ams, Amsterdam; CPA, cyproterone acetate; E, estradiol; G, injected, intrauterine, or oral progestin; G_0, progestin at baseline; Ghe, Ghent; IM, intramuscular; T, testosterone.

a Age is highest in the E transdermal + CPA group of transgender women (Mann–Whitney U test, *P* < .05). Previously published by Bøgehave et al. [25], [26].

b Shifting over time between oral and transdermal therapy (in transgender women) or between testosterone undecanoate and ester and gel (in transgender men).

### 2.2 Blood sampling

Venous blood was collected from the antecubital vein at baseline and after 12 months of GAHT after seated rest. Samples were drawn into 0.105/0.109 sodium citrate tubes. Platelet-poor plasma was obtained by centrifugation (1800 × *g*, 10 minutes) and stored at −80 °C until analysis. After thawing at 37 °C, samples were analyzed within 1 hour, with paired samples from each participant run in the same series.

### 2.3 Biochemical analysis

Fibrinogen γ’ was measured in citrate plasma using an in-house ELISA developed by Pedersen et al. [29]. The assay employs a mouse anti-human fibrinogen γ’ monoclonal capture antibody and a polyclonal rabbit anti-human fibrinogen detection antibody (Dako). A fibrinogen γ’ calibrator (P2 Fib, Enzyme Research Laboratories) was used for quantification. Precision of the assay was 7% and 3% for inter- and intra-assay coefficients of variation, respectively. Plasma analyses were performed in 245 transgender women and 318 transgender men, reflecting the availability of citrate plasma.

Total fibrinogen concentrations were measured by nephelometry and previously reported [25], and relative fibrinogen γ’ levels were calculated as the ratio between fibrinogen γ’ and total fibrinogen. Previously reported E and T concentrations [26] were measured using immunoassays, with liquid chromatography–tandem mass spectrometry also used for E after July 2014 [28].

### 2.4 Statistical analysis

The measured fibrinogen variables were normally distributed and were compared at baseline and 12 months with a paired *t*-test. Between-group differences were assessed with ANOVA at baseline and with linear regression at 12 months, adjusting for baseline values and, in transgender women, also for age. Between-group differences in absolute changes (Δ 0-12 months) were assessed using linear regression. Robust standard errors were applied when model assumptions were not met. Significant overall effects were followed by Bonferroni-corrected pairwise comparisons. Results are reported as mean with 95% CIs, and *P* < .05 was considered statistically significant. Associations between changes in previously published circulating hormone levels (E and T) [26] and changes in absolute and relative fibrinogen γ’ and total fibrinogen were assessed using Spearman’s rank correlation. Analyses were performed in Stata 18 (StataCorp). The analysis reflects a per-protocol approach, as fibrinogen γ’ was only measured in participants who completed 12 months of GAHT.

## 3 Results and Discussion

Among individuals receiving feminizing GAHT, fibrinogen γ’ increased significantly from baseline to 12 months in the combined cohort (E total), paralleling the previously reported rise in total fibrinogen [25], whereas the relative fibrinogen γ’ levels (ratio of fibrinogen γ’ to total fibrinogen) decreased (Table 2 and Figure). Subgroup analysis showed a significant increase in fibrinogen γ’ in the E oral + CPA subgroup. Relative levels decreased across all feminizing GAHT regimens, reaching statistical significance in the E oral + CPA subgroup. We observed between-group differences after 12 months for fibrinogen γ’ (*P* = .02), due to higher values in the E oral + CPA group compared with the E transdermal + CPA group (Δ fibrinogen γ’: 62 μg/mL [95% CI: 10; 113], *P* = .01) (Table 2). This between-group difference was also reflected in absolute changes (Δ 0-12 months), where both fibrinogen γ’ (*P* = .002) and total fibrinogen (*P* = .001) increased more in the oral + CPA group than in the transdermal E + CPA group (Δ fibrinogen γ’: 67 μg/mL [95% CI: 22; 113], *P* = .001; Δ total fibrinogen: 298 μg/mL [95% CI: 106; 489], *P* = .001), whereas relative levels did not differ between groups (Figure).

**Table 2:** Fibrinogen variables in transgender women before and 12 months after gender-affirming hormone therapy.

Variable	0 months	12 months	*P*
Fibrinogen γ’ (μg/mL)			
E total (*n* = 245)	588 (566; 610)	628 (605; 651)	**<.001**
E oral + CPA (*n* = 161)	571 (546; 596)	632 (604; 659)	**<.001**
E transdermal + CPA (*n* = 68)	628 (581; 675)	622 (575; 668)^a^	.72
E oral/transdermal + CPA (*n* = 16)	592 (495; 690)	616 (531; 700)	.27
Total fibrinogen (μg/mL)^b^			
E total (*n* = 243)	2624 (2551; 2697)	2860 (2789; 2930)	**<.001**
E oral + CPA (*n* = 161)	2501 (2417; 2584)	2823 (2732; 2914)	**<.001**
E transdermal + CPA (*n* = 66)	2916 (2768; 3065)^c^	2941 (2817; 3066)	.72
E oral/transdermal + CPA (*n* = 16)	2659 (2445; 2874)	2889 (2672; 3106)	**.03**
Fibrinogen γ’ ratio			
E total (*n* = 243)	0.227 (0.220; 0.234)	0.221 (0.214; 0.227)	**.003**
E oral + CPA (*n* = 161)	0.231 (0.223; 0.240)	0.225 (0.217; 0.233)	**.02**
E transdermal + CPA (*n* = 66)	0.216 (0.203; 0.230)	0.211 (0.198; 0.225)	.20
E oral/transdermal + CPA (*n* = 16)	0.226 (0.187; 0.264)	0.216 (0.185; 0.246)	.23

Values presented as mean (95% CI) were compared at 0 and 12 months (within-group changes) with a paired *t*-test. Between-group comparisons (between gender-affirming hormone therapy administration forms) at 0 months were performed with an ANOVA. Between-group comparisons at 12 months were performed with a linear regression analysis, adjusted for baseline values (0 months) and age. When significant between-group effects were observed in the ANOVA or linear regression analysis, pairwise comparisons were performed with the Bonferroni test. Bold text indicates statistically significant results (*P* < .05).

ANOVA, analysis of variance; CPA, cyproterone acetate; E, estradiol; oral/transdermal, oral and transdermal estradiol shifting over time.

a Significantly lower than E oral + CPA (*P* < .05).

b Between-group comparisons at 12 months were performed with a multivariate linear regression analysis with log-transformed outcome variables to meet model assumptions.

c Significantly higher than E oral + CPA (*P* < .001).

**Figure:**
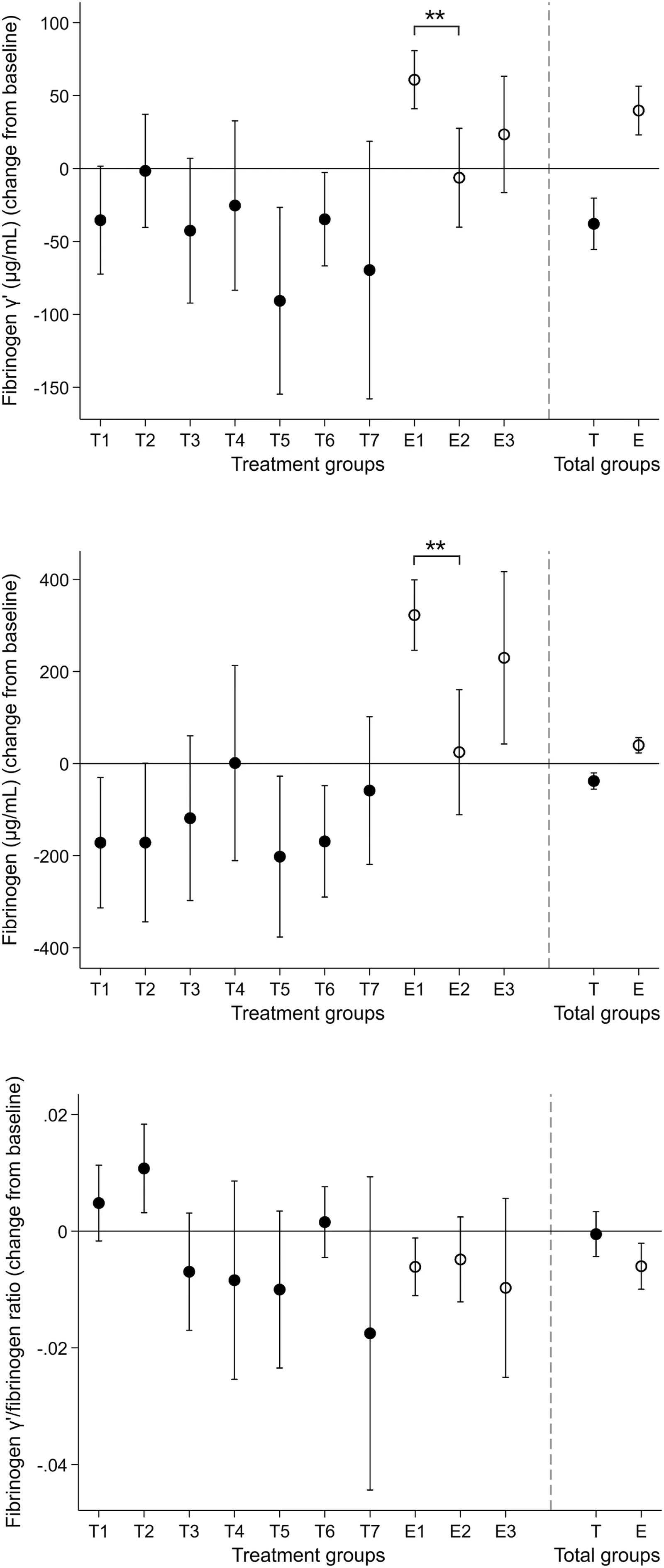
Absolute changes in fibrinogen variables from baseline to 12 months in transgender women and transgender men according to GAHT regimen. Results are presented as mean with 95% CI. ○ feminizing GAHT, • masculinizing GAHT. Changes (0–12 months) were compared between GAHT administration forms using linear regression analysis (with robust standard errors for total fibrinogen to meet model assumptions). When significant between-group effects were observed, pairwise comparisons were performed with the Bonferroni test. ⁎⁎*P* < .01. T1: T IM undecanoate; T2: T IM ester; T3: T transdermal; T4: T mixed; T5: T mixed + G; T6: G_0 + T mixed; T7: G_0 + T mixed + G; E1: E oral + CPA; E2: E transdermal + CPA; E3: E oral/transdermal + CPA (group abbreviations are defined in Table 1). CPA, cyproterone acetate; E, estradiol; G, injected, intrauterine, or oral progestin; G_0, progestin at baseline; GAHT, gender-affirming hormone therapy; IM, intramuscular; T, testosterone; T mixed, testosterone undecanoate or ester or gel shifting over time.

In individuals receiving masculinizing GAHT, fibrinogen γ’ decreased from baseline to 12 months for all masculinizing GAHT groups combined (T total), mirroring the previously reported decrease in total fibrinogen [25], while relative fibrinogen γ’ levels remained unchanged (Table 3 and Figure). At the subgroup level, fibrinogen γ’ decreased across all masculinizing GAHT regimens, reaching statistical significance in the T mixed + G group and in participants receiving G until study start (G_0 + T mixed). For the relative fibrinogen γ’ levels, no clear directional pattern emerged across GAHT regimens. We observed no significant between-group differences at 12 months or for changes (Δ 0-12 months).

**Table 3:** Fibrinogen variables in transgender men before and 12 months after gender-affirming hormone therapy.

Variable	0 months	12 months	*P*
Fibrinogen γ’ (μg/mL)			
T total (*n* = 318)	698 (675; 722)	660 (640; 680)	**<.001**
T IM undecanoate (*n* = 83)	666 (619; 712)	630 (596; 665)	.06
T IM ester (*n* = 47)	682 (630; 734)	680 (624; 737)	.93
T transdermal (*n* = 40)	640 (575; 705)	598 (545; 650)	.10
T mixed (*n* = 30)	713 (640; 787)	688 (617; 759)	.40
T mixed + G (*n* = 33)	820 (720; 920)^a^	730 (663; 796)	**.01**
G_0 + T mixed (*n* = 67)	697 (652; 743)	663 (618; 707)	**.04**
G_0 + T mixed + G (*n* = 18)	776 (657; 895)	706 (607; 805)	.14
Total fibrinogen (μg/mL)^b^			
T total (*n* = 316)	3011 (2940; 3083)	2866 (2801; 2932)	**<.001**
T IM undecanoate (*n* = 83)	2987 (2864; 3110)	2815 (2697; 2933)	**.02**
T IM ester (*n* = 47)	3097 (2877; 3316)	2925 (2723; 3128)	.06
T transdermal (*n* = 39)	2844 (2617; 3071)	2725 (2569; 2881)	.20
T mixed (*n* = 30)	2955 (2756; 3153)	2956 (2780; 3132)	.99
T mixed + G (*n* = 33)	3114 (2857; 3371)	2912 (2687; 3167)	**.03**
G_0 + T mixed (*n* = 66)	3002 (2849; 3153)	2833 (2677; 2990)	**.01**
G_0 + T mixed + G (*n* = 18)	3201 (2837; 3565)	3142 (2818; 3467)	.48
Fibrinogen γ’ ratio			
T total (*n* = 316)	0.233 (0.227; 0.240)	0.233 (0.227; 0.239)	.80
T IM undecanoate (*n* = 83)	0.222 (0.211; 0.233)	0.227 (0.215; 0.238)	.15
T IM ester (*n* = 47)	0.224 (0.210; 0.239)	0.235 (0.219; 0.251)	**.01**
T transdermal (*n* = 39)	0.229 (0.211; 0.246)	0.222 (0.206; 0.238)	.18
T mixed (*n* = 30)	0.243 (0.221; 0.265)	0.235 (0.212; 0.258)	.34
T mixed + G (*n* = 33)	0.263 (0.241; 0.284)^c^	0.253 (0.235; 0.271)	.16
G_0 + T mixed (*n* = 66)	0.235 (0.223; 0.248)	0.237 (0.224; 0.249)	.62
G_0 + T mixed + G (*n* = 18)	0.244 (0.214; 0.274)	0.227 (0.199; 0.254)	.22

Values presented as mean (95% CI) were compared at 0 and 12 months (within-group changes) with a paired t-test. Between-group comparisons (between GAHT administration forms) at 0 months were performed with an ANOVA. Between-group comparisons at 12 months were performed with a linear regression analysis, adjusted for baseline values (0 months). When significant between-group effects were observed in the ANOVA or linear regression analysis, pairwise comparisons were performed with the Bonferroni test. Bold text indicates statistically significant results (*P* < .05).

ANOVA, analysis of variance; G, injected, intrauterine, or oral progestin; G_0, progestin at baseline; IM, intramuscular; T, testosterone; T mixed, testosterone undecanoate or ester or gel shifting over time.

a Significantly higher than T IM undecanoate and T transdermal (*P* < .01).

b Between-group comparisons at 12 months were performed with a multivariate linear regression analysis with log-transformed outcome variable to meet model assumptions.

c Significantly higher than T IM undecanoate and T IM ester (*P* < .05).

Changes in E and T levels did not correlate with changes in absolute or relative fibrinogen γ’ or total fibrinogen, neither in the total group of individuals receiving feminizing (ρ = −.120 to .097, all *P* > .05) or masculinizing GAHT (ρ = −.032 to .085, all *P* > .05) nor in the oral, intramuscular, and transdermal subgroups (results not shown). Variability related to the timing of blood sampling relative to hormone administration and the use of different assays may have limited the ability to detect such associations.

The present study did not confirm our hypothesis that fibrinogen γ’ levels decrease during feminizing GAHT and increase during masculinizing GAHT. However, we demonstrated that GAHT modulates fibrinogen γ’ in a direction that differs between feminizing and masculinizing GAHT. As fibrinogen γ’ is generated through alternative splicing of the fibrinogen γ-chain, these findings suggest sex hormone–dependent regulation of this fibrinogen isoform. Estrogen exposure, particularly oral administration, is known to influence hepatic protein synthesis, consistent with the observed increases in total fibrinogen and fibrinogen γ’ in individuals using feminizing GAHT. Similar directional changes across feminizing regimens suggest a shared estrogen-related effect, while differences in magnitude may reflect route-dependent hepatic exposure and suggest a less pronounced effect of transdermal E on absolute fibrinogen γ’ levels. The reduction in relative fibrinogen γ’ levels indicates that total fibrinogen increased more than fibrinogen γ’ levels during feminizing GAHT, suggesting differential regulation of fibrinogen γ’ and total fibrinogen synthesis. Whether this reflects altered splicing or broader changes in fibrinogen synthesis cannot be determined from the present data. Conversely, the preserved relative fibrinogen γ’ levels in individuals using masculinizing GAHT indicate that reductions in fibrinogen γ’ largely parallel changes in total fibrinogen. To our knowledge, the effects of sex hormones (including oral contraceptives) on fibrinogen γ’ have not been studied in cisgender populations.

In individuals receiving feminizing GAHT, epidemiologic studies have most consistently reported an increased risk of venous thrombosis during feminizing GAHT, although increased arterial thrombotic risk has also been reported [20], [22]. Observational studies have demonstrated that lower absolute and relative fibrinogen γ’ levels are associated with increased risk of venous thrombosis [10], [11], although findings have not been consistent [9], [30]. In the present study, fibrinogen γ’ increased after feminizing GAHT, whereas the relative levels of fibrinogen γ’ decreased. Fibrinogen γ’ influences both fibrin clot structure and anticoagulant pathways, contributing to a complex role in hemostasis [12], [18], [19]. Increased fibrinogen γ’ levels have been associated with denser and more lysis-resistant fibrin clots [12], [15], [16], [17], whereas fibrinogen γ’ also binds thrombin with high affinity, inhibits thrombin-mediated activation of coagulation factor V, and enhances sensitivity to activated protein C [18], [19]. Consequently, a lower relative proportion of fibrinogen γ’ can reduce fibrinogen γ’-mediated anticoagulant effects despite increasing fibrinogen γ’ concentrations. This may be relevant to venous thrombosis, as resistance to activated protein C is one of the most common risk factors for venous thrombosis [19]. The clinical implications of these opposing changes remain uncertain, particularly given previous associations between elevated absolute fibrinogen γ’ levels and arterial thrombotic disease [6], [7], [8], [9]. Notably, the reduction in relative fibrinogen γ’ levels occurred alongside increased fibrin formation and impaired clot lysis previously observed in this cohort [25], suggesting remodeling of fibrin clot characteristics during feminizing GAHT. Taken together, these findings suggest that the reduced relative fibrinogen γ’ levels may be relevant to the increased venous thrombotic risk observed during feminizing GAHT.

Fibrinogen γ’ and total fibrinogen decreased in parallel across masculinizing GAHT regimens, with unchanged relative fibrinogen γ’ levels, suggesting that masculinizing GAHT does not selectively modulate the synthesis of fibrinogen γ’. Epidemiologic data indicate an increased risk of arterial thrombotic events during masculinizing GAHT [22]. In contrast to previous studies linking elevated fibrinogen γ’ levels to arterial thrombotic disease [6], [7], [8], [9], fibrinogen γ’ levels decreased after masculinizing GAHT and were accompanied by reduced fibrin formation and enhanced clot lysis previously observed in this cohort [25]. These findings do not support a fibrinogen γ’-mediated mechanism underlying the increased arterial risk associated with masculinizing GAHT. Instead, arterial risk is likely multifactorial, with contributions from androgen-related effects on lipids, hematocrit, and blood pressure [26].

Relative fibrinogen γ’ levels in the present cohort (22%-23% at baseline) were slightly higher than the 19% previously observed in healthy blood donors using the same assay [3]. Differences in population characteristics, including age, body mass index, smoking, and medication use, may contribute to this difference. In contrast, the difference from the 12% to 15% reported in some studies using another antibody [5] may, at least in part, be assay-dependent [29].

A major strength of this study is the relatively large cohort of transgender and gender-diverse persons with paired samples obtained before and after 12 months of exposure to different GAHT regimens, although there may have been limited statistical power to detect smaller differences, particularly in subgroup analyses. Fibrinogen γ’ was not defined as a primary outcome in the original study [27], and the present study should be regarded as a pilot study. A limitation is that we did not include a control group of healthy cisgender individuals followed over 12 months, but Pedersen et al. [3] previously demonstrated that both absolute and relative levels of fibrinogen γ’ do not change during 24 months in cisgender persons. Besides, ethnicity data were not available, which may limit the generalizability of our findings across different populations. Another limitation is that all transgender women used CPA, and our results cannot be generalized to other types of antiandrogens.

Overall, feminizing and masculinizing GAHT for 12 months had opposite effects on fibrinogen γ’ levels. Feminizing GAHT increased absolute fibrinogen γ’ levels but reduced relative levels, whereas masculinizing GAHT decreased absolute levels without affecting relative fibrinogen γ’ levels. The reduction in relative fibrinogen γ’ levels during feminizing GAHT may be relevant to the increased venous thrombotic risk associated with feminizing GAHT, whereas the changes observed during masculinizing GAHT do not support a fibrinogen γ’-mediated explanation for the increased arterial thrombotic risk associated with masculinizing GAHT.
